# Impact of High-Fat Diet-induced Metabolic Dysfunction-associated Steatotic Liver Disease on Heart, Kidney, and Skeletal Muscle Metabolomes in Wild-Type Mice

**DOI:** 10.1021/acs.jproteome.5c00040

**Published:** 2025-04-13

**Authors:** João G. Silva, Ludgero Tavares, Getachew D. Belew, João A. Rodrigues, Rita Araújo, Ana M. Gil, John G. Jones

**Affiliations:** † Institute for Interdisciplinary Research (III-UC), Centre for Innovative Biomedicine and Biotechnology (CIBB), Metabolism, Aging and Disease, 37829 University of Coimbra, Cantanhede 3060-197, Portugal; ‡ Institute for Interdisciplinary Research (III-UC), Doctoral Programme in Experimental Biology and Biomedicine (PDBEB), University of Coimbra, Coimbra 3030-789, Portugal; § University School Vasco da Gama (EUVG), Vasco da Gama Research Center (CIVG), Coimbra 3020-210, Portugal; ∥ Department of Biomedical Sciences, Heritage College of Osteopathic Medicine, Ohio University, Athens, Ohio 45701, United States; ⊥ Department of Chemistry and CICECO-Aveiro Institute of Materials, University of Aveiro, Aveiro 3810-193, Portugal

**Keywords:** metabolic dysfunction-associated steatotic liver disease, metabolomics, extrahepatic tissues, metabolic syndrome, nuclear magnetic resonance, tissue extracts

## Abstract

**Background:** Metabolic dysfunction-associated steatotic liver disease (MASLD) can be recapitulated in mice fed a high-fat diet. The development of MASLD and the diet *per se* can both perturb metabolism in key extrahepatic tissues such as the heart, kidney, and skeletal muscle. To date, these alterations have not been well described in this animal model of diet-induced MASLD. **Methodology:** Male C57BL/6J mice were fed either standard (SC, *n* = 12) or high-fat chow (HF, *n* = 11) for 18 weeks. Metabolites were extracted from the heart, kidney, and skeletal muscle and analyzed by ^1^H nuclear magnetic resonance (NMR) spectroscopy, along with multivariate and univariate statistical analyses. **Results:** Kidney metabolite profiles exhibited the largest differences between HF and SC diets, followed by those of skeletal muscle and then the heart. Some alterations were common across all tissues, namely decreased trimethylamine and elevated levels of linoleic acid and polyunsaturated fatty acids in HF compared to SC (*p* < 0.05 for all three metabolites). Overall, the metabolite variations were consistent with shifts in carbohydrate and lipid substrate selection for oxidation, increased tissue stress in the heart and kidneys, and altered choline metabolism. These findings may serve as additional important descriptors of MASLD onset and progression.

## Graphical abstract

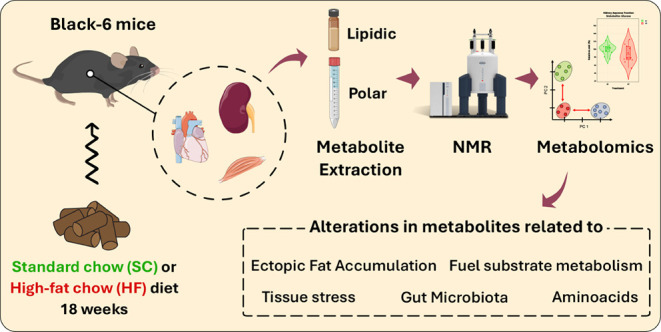

## Introduction

Metabolic dysfunction-associated steatotic liver disease (MASLD), formerly known as non-alcoholic fatty liver disease (NAFLD), is currently one of the most prevalent noncommunicable diseases worldwide.^1^ As of 2023, Younossi et al. estimated that 35% of the world’s population is afflicted by this condition.^2^ While MASLD development and progression include intrinsic risk factors such as sex, age, ethnicity, and genetics, there is broad consensus that overconsumption of highly processed, energy-dense foods rich in fat and sugar are important drivers of the disease.^3–5^ Together with sedentary lifestyles, such habits explain the established high incidence of MASLD in urbanized Western countries^6,7^ as well as its steep increase in developing countries that aspire to Westernized economies and lifestyles.^2^

The manifestation of MASLD reflects a surfeit of neutral lipids—mainly triglycerides (TG)—that accumulate in hepatocytes and other liver cell types, which normally contain residual lipid levels of ≤20 mg/g wet weight.^8^ Indeed, the current definition of MASLD in humans involves a hepatic TG content exceeding 55.6 mg/g of wet liver weight, established after the first population-wide magnetic resonance survey of liver TG content.^9^ MASLD pathophysiology is characterized by two important aspects: (a) specific alterations in hepatic lipid appearance and clearance fluxes^10^ and (b) secondary effects on other nutrient fluxes—notably carbohydrate disposal and oxidation.^11,12^ One of the best-characterized secondary effects is the disruption of insulin signaling by so-called lipotoxic lipid species, such as ceramides and diacylglycerols, leading to an insulin-resistant state.^13,14^ The onset and progression of MASLD are also tightly linked with a number of extrahepatic comorbidities that affect other major metabolic organs, namely the skeletal muscle, heart, and kidney. Studies have shown MASLD to be an independent risk factor for sarcopenia,^15,16^ coronary artery disease^17,18^ and chronic kidney disease.^19,20^

In rodent models, MASLD is most commonly induced by feeding a high-fat diet over a period ranging from about 8–36 weeks.^21^ Under these conditions, the typical disease profile resembles that of simple or early MASLD in humans: a robust increase in hepatic TG concentrations but with limited indications of fibrosis or other hepatic injury that characterize the progression of simple MASLD to metabolic-associated steatohepatitis (MASH).^22^ To date, while hepatic alterations have been well characterized in these models, to the best of our knowledge, the metabolic profiles of skeletal muscle, heart, and kidney are less well described. MASLD can directly influence the physiological states of extrahepatic tissues, for example, via increased secretion of certain hepatokines^23^ and proinflammatory agents,^24^ and the development of insulin resistance. Insulin resistance *per se* can also independently provoke metabolic perturbations in a wide range of extrahepatic sites, including the heart,^25^ skeletal muscle^26^ and kidney.^27^ These can, in turn, promote and accelerate MASLD, thereby generating a cycle where control of glucose and lipid metabolism is disrupted system-wide, and a cluster of significant extrahepatic comorbidities arises alongside the development of MASLD itself.^28^

On this basis, a better understanding of the metabolic status of tissues such as the heart, skeletal muscle, and kidney in diet-induced rodent MASLD models can provide a more holistic description of the disease phenotype. Moreover, given the well-known impacts of MASLD on the metabolic states of extrahepatic tissues in humans, it is important to determine to what extent these are recapitulated in commonly used rodent models of MASLD. To this end, an unbiased and systematic metabolic profiling study of selected extrahepatic tissues, namely the heart, kidney, and skeletal muscle, is an informative first step in characterizing their metabolic status in rodent models of MASLD. Nuclear magnetic resonance (NMR) spectroscopy metabolomics of tissue extracts provides an acceptable balance between the level of metabolome coverage of these different tissues, high sample throughput, and reproducibility in both sample preparation and data acquisition.^29–31^ Here, we report on an NMR metabolomics characterization of the effects of MASLD induced by a high-fat (HF) diet on C57BL/6 mice, compared with standard chow (SC), as viewed through the metabolic responses of the heart, kidney, and skeletal muscle. While these tissues are highly involved in off-target MASLD effects, to date, they have been largely overlooked in preclinical MASLD models.

## Material and Methods

### Animal Studies

Figure 1 presents an overview of the workflow followed in this study. The study was approved by the University of Coimbra Ethics Committee on Animal Studies (ORBEA) and the Portuguese National Authority for Animal Health (DGAV) (No. 0421/000/000/2017, 5/6/2017). All animal procedures were performed in full accordance with DGAV guidelines and European regulations (European Union Directive 2010/63/EU).

**Figure 1:**
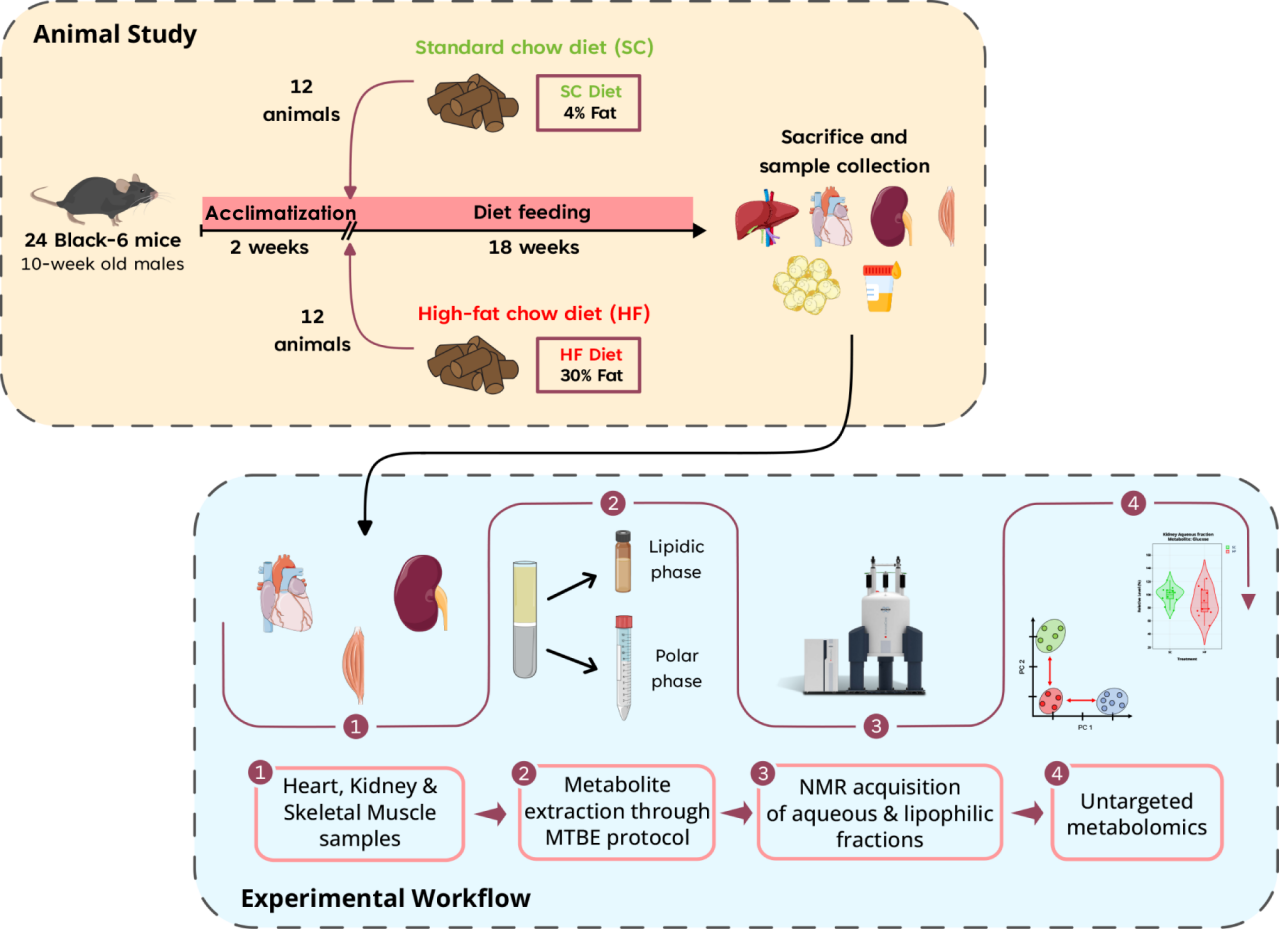
Experimental design displaying both the animal study time timeline and the sample handling workflow. From black-6 mice, fed either standard chow or high-fat diets for 18 weeks, the heart, kidney, and skeletal muscle tissues were collected and subsequently exposed to a metabolite extraction method. The resulting polar and lipidic phases were acquired with untargeted ^1^H NMR for metabolome analysis. Some elements were adapted from Bioicons and Scidraw and licensed under a Creative Commons Attribution (CC-BY) license.

Twenty-four adult male C57BL/6J mice were obtained from Charles River Laboratories (Barcelona, Spain; RRID: IMSR_JAX:000664) at 8 weeks of age and were housed at the University of Coimbra UC Biotech Bioterium. The mice were kept in a well-ventilated environment with a 12-h light/dark cycle. Upon arrival at the Bioterium, the mice were randomly assigned to cages with four mice per cage and given a two-week adaptation period with free access to water and standard chow. After acclimatization, 12 of the mice were provided with high-fat chow (41% carbohydrate, 30% fat, 25% protein, and 4% ash; Mucedola s.r.l. 223425) (HF group), while the remaining mice were kept on standard chow (73% carbohydrate, 4% fat, 19% protein, and 4% ash; Mucedola s.r.l. 223426) (SC group) during the following 18 weeks. Mice were weighed at the start of the 18-week period and then every 2 weeks until the end of the experiment. During the feeding experiment, one animal from the HF group was euthanized due to a subcutaneous mass in the peri-preputial region, suggesting a possible inflammation/infection of the preputial glands, and as such was not included in the final data. A heart sample from another HF mouse was lost in storage; hence, the final number of HF animals studied through cardiac tissue analysis was 10. On the final morning, all mice were deeply anesthetized with ketamine/xylazine and sacrificed by cardiac puncture. Whole organs (liver, heart, kidney) and hind limb skeletal muscle tissue were freeze-clamped and stored at −80 °C until further analysis. The MASLD staging profile in these same mice was previously reported as part of a larger study,^32^ through liver histology, as well as measurements of hepatic TG content and whole-body adiposity.

### Tissue Metabolites Extraction

Mice whole hearts and kidneys, along with sections of hind limb skeletal muscle (all maintained on dry ice), were submerged in 500 μL of ice-cold methanol, pulverized using a tissue homogenizer (IKA ULTRA-TURRAX), and then kept on ice. Polar and nonpolar metabolites were obtained using the methyl *tert*-butyl ether (MTBE) extraction protocol, as previously described.^33,34^ To the homogenized tissue, 4.6 mL of ice-cold methanol/g wet weight were added, followed by vortex mixing. Subsequently, 15.4 mL of MTBE/g wet weight were added and vigorously mixed at room temperature. The mixture was then centrifuged for 10 min at 13000 *g* at room temperature, followed by the addition of 4 mL of water/g wet weight to the supernatant. After a 10-min rest, the mixture was centrifuged at 1000 *g* for 10 min at room temperature, and the lipophilic and aqueous layers were collected into separate vials. The aqueous fractions were lyophilized and stored at −80 °C until NMR analysis, while the lipophilic fractions were kept in the dark and air-dried under a hood for 24 h or until fully dried, followed by storage at −20 °C (for a maximum of 1 year) until NMR analysis.

### NMR Spectroscopy

^1^H NMR spectra of each sample were acquired using a Bruker Avance III spectrometer operating at 500.13 MHz for proton detection (at 25 °C), employing a Nuclear Overhauser effect 1D pulse sequence (*noesypr1d*, Bruker library) for aqueous extracts or a standard 1D pulse sequence (*zg*, Bruker library) for lipophilic extracts, both with a 7002.801 Hz spectral width. For aqueous extracts, each sample was resuspended in 600 μL of ^2^H_2_O phosphate buffer (0.1 M Na_2_HPO_4_/NaH_2_PO_4_) containing 0.1 mM sodium 3-(trimethylsilyl)­propionate-2,2,3,3-*d*_4_ (TSP) for chemical shift referencing, and the pH was adjusted to 7.4 using deuterated hydrochloric acid and deuterated potassium hydroxide. Regarding the lipophilic extracts, each sample was resuspended in 600 μL of 99.98% deuterated chloroform containing 0.24 mM pyrazine for chemical shift referencing. From each sample, a 550 μL aliquot was transferred to a 5 mm diameter NMR tube. Data were collected using 256 scans, with a 4 s relaxation delay (d1), 2.33 s acquisition time (corresponding to 32k data points), and 0.1 s mixing time. For selected samples, additional 2D TOCSY acquisitions were performed to confirm metabolite assignments. NMR spectra were analyzed using Bruker Topspin 4.1 software (Bruker, Germany. RRID: SCR_014227). Prior to Fourier transformation, free induction decays were processed with 0.3 Hz line broadening and zero-filled to 131,072 and 65,536 data points for aqueous and lipophilic samples, respectively. Chemical shifts were referenced to the TSP resonance at δ 0.0 for aqueous samples and to the pyrazine signal at δ 8.6 for lipophilic samples. Peak assignments were confirmed using literature, 2D NMR spectra of selected samples, and the following spectral databases: ChenomX NMR Suite 8.3 (ChenomX, Canada. RRID: SCR_014682) and the human metabolome database (HMDB) version 5.0 (RRID: SCR_007712).^35^ For the assignment of creatine (Cr) and phosphocreatine (PCr), whose chemical shifts are highly pH-sensitive, selected samples were spiked with commercial standards prepared in the sample buffer and adjusted to pH 7.4. Four of the aqueous extracts of skeletal muscle (3 samples from the SC group and 1 from the HF group) presented white cloudy appearances, with altered spectra suggestive of contamination, and thus were excluded from analysis.

### Data Processing and Statistical Analyses

The spectral regions corresponding to the resonances of water (δ 4.70–5.00), residual methanol (δ 3.34–3.37) in aqueous extracts, and chloroform (δ 7.03–7.49) and pyrazine (δ 8.50–8.80) in lipophilic extracts were excluded from the analysis (using Amix 3.9.15, Bruker, Germany. RRID: SCR_025468). Alignment of signals was performed using the recursive segment-wise peak alignment script in MATLAB R2019b (MathWorks Inc., Natick, Massachusetts, USA. RRID: SCR_001622). NMR signal intensities were normalized by total spectral area for each sample. Principal component analysis (PCA) and partial least-squares discriminant analysis (PLS-DA) were performed in a SIMCA-P 18 instrument (Umetrics, Sweden. RRID: SCR_014688) after Pareto or Unit Variation (UV) scaling. Data were extracted from SIMCA and plotted using Python 3.11 (RRID: SCR_008394) with the Plotly package for visualization. PLS-DA loading weights were obtained by multiplying each variable by its standard deviation and coloring according to variable importance to projection (VIP) (Python, Matplotlib, and Plotly packages). PLS-DA model performance was assessed by *R* ^2^ scores (fit power), *Q* ^2^ values (predictive power), and through permutation tests (500 permutations) in SIMCA. In addition, two validation tools were used: Monte Carlo cross validation (MCCV), with seven blocks and 500 permutations via an in-house script, providing *Q* ^2^ distributions (cross-validation prediction power), classification rates (CR), specificity, and sensitivity; and k-fold cross-validation area under the receiver operator characteristic (AUROC) analysis, performed in Python, with a 5 k-fold split using the random forest classifier method and 100 permutations for each k-fold (scikit-learn and Matplotlib packages). Univariate analysis was carried out upon peak integration (Amix 3.19.15) and normalization. Value distributions were tested for normality using the Shapiro-Wilk test, and effect size values were computed.^36^ For normal distributions, a Student’s *t*-test with a Welch correction was applied, while variables that failed normality were assessed using the nonparametric Mann–Whitney test. All *p*-value results were adjusted by false discovery rate (FDR) correction for multiple comparisons, based on the Benjamini and Hochberg method.^37^ All tests were performed in Python (SciPy, scikit-posthocs, and statsmodels packages for calculations, and the Plotly package for visualization). Statistical significance was defined as confidence α = 0.95 and was indicated in figures as * for *p*-value <0.05, ** for *p*-value <0.01, and *** for *p*-value <0.001. Data sets were plotted as heatmaps representing fold-change between diet groups (GraphPad Prism 9 Software, Boston, Massachusetts, USA. RRID: SCR_002798).

In addition to standard metabolomics analysis, precise quantitative analysis of lipid profiles was carried out, as previously described.^38,39^ For that purpose, NMR spectra of a subset of lipophilic extract samples were acquired with a d1 of 15 s to achieve conditions of complete longitudinal relaxation times (*T*_1_). The percentages of saturated (SFA), polyunsaturated (PUFA), monounsaturated (MUFA), and ω-3 FA were calculated as fractions of total FA in each tissue. For quantification of total FA levels, total FA CH_3_ concentration was assessed by summing the corrected FA-CH_3_ signals relative to that of pyrazine and normalizing to tissue wet weight. Statistical significance analysis for the lipidome was then performed as described for univariate analysis.

## Results

### Mouse Model MASLD Profile

The data presented are part of a previously published study^32^ and are included in the Supporting Information to provide a description of the MASLD phenotype that developed following 18 weeks of high-fat feeding. In summary, HF mice gained weight at a faster rate compared to SC mice (Figure S1a,b), while total body adiposity and total hepatic TG end levels were also significantly higher in HF animals (Figure S1d,e). Liver histology was assessed by a pathologist (Figure S1c) and revealed a pattern of moderate steatosis and mild ballooning, with no significant indication of fibrosis. Thus, the MASLD phenotype resembles that of simple MASLD in humans and is typical of the initial stages of disease.

### ^1^H NMR Metabolic Profiles of Mouse Kidney, Heart, and Skeletal Muscle

Figure 2 shows representative ^1^H NMR spectra for kidney aqueous and lipophilic extracts (Figures 2c,d, respectively) corresponding to mice fed SC (Figure 2a) and HF (Figure 2b) diets. Figures S2 and S3 display the corresponding spectra obtained from heart and skeletal muscle extracts, respectively. Table S1 presents the overall list of assigned peaks across the 3 tissues. While published information on the ^1^H NMR spectra of water-soluble metabolites for these tissues is rather limited, by cross-checking various databases with data from published studies under different conditions, it was possible to assign the vast majority of signals in the spectra obtained from the three tissues.^29,31,40–44^ Overall, the highest-intensity signals observed in all tissues were typically from lactate, alanine, taurine, and creatine. Among the weaker signals, niacinamide, inosine monophosphate (IMP), trimethylamine (TMA), dimethylamine (DMA), and ethanolamine were identified for the first time in such tissues. Skeletal muscle aqueous extracts had the highest number of identified signals, followed by kidney and heart (70, 57, and 39, respectively) (Table S1). The spectra of lipophilic extracts presented signals arising mainly from FA moieties of several families of neutral and charged lipids (Figures 2c,d, S2c,d, and FS3c,d), consistent with previous reports.^38,45–48^ Overall, around 30 signals were identified in the spectra of lipid extracts (33 in the heart, 33 in the kidney, and 30 in skeletal muscle).

**Figure 2:**
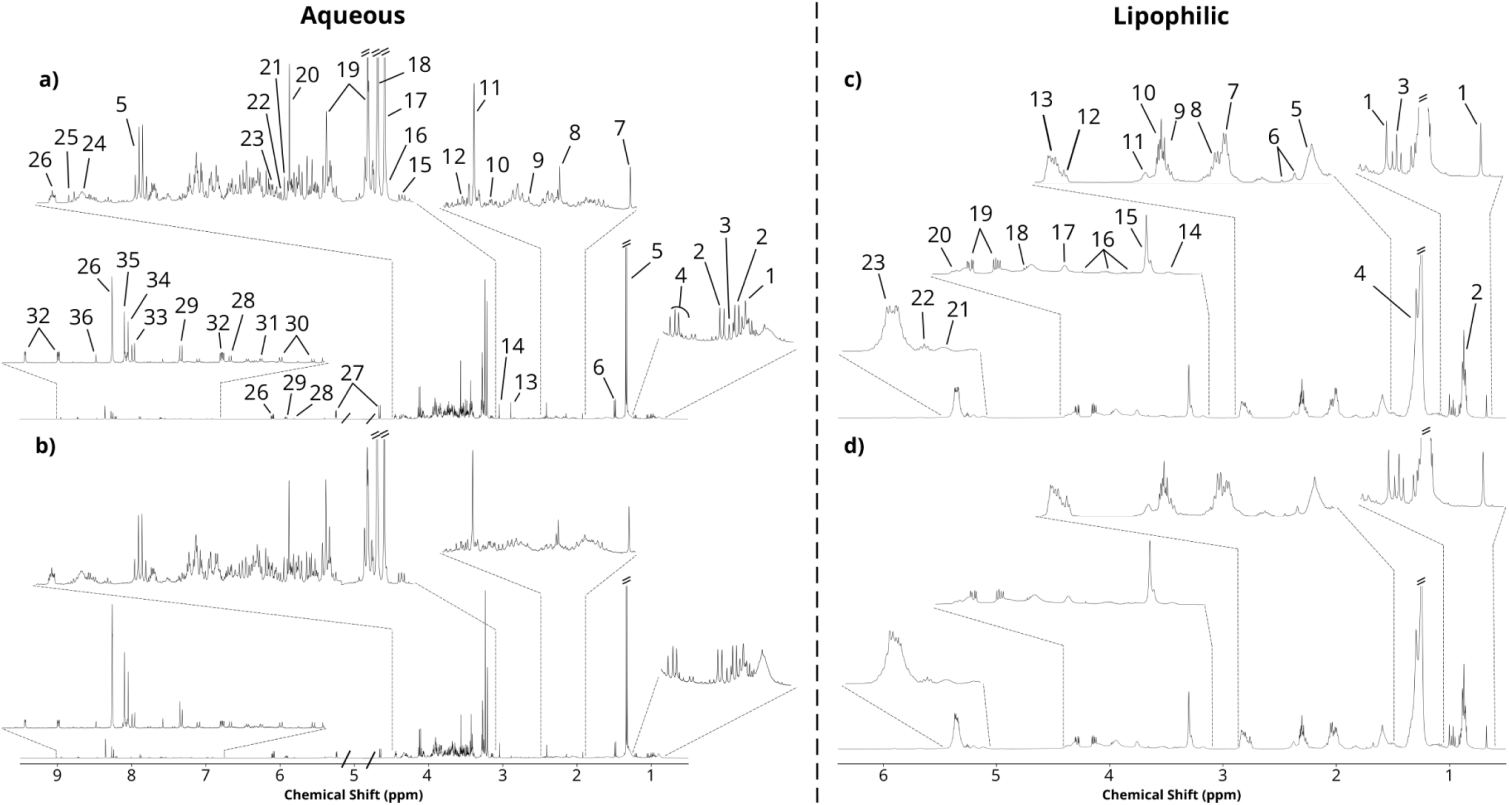
Typical 500 MHz ^1^H NMR spectra obtained for mice kidney extracts. (a,b) Aqueous extracts from (a) SC diet and (b) HF chow diet. Vertical intensity is matched between the spectra. Main assignments are as follows: 1. leucine; 2. valine; 3. isoleucine; 4. 3-hydroxybutyrate and ethanol; 5. lactate; 6. alanine; 7. acetate; 8. methionine; 9. acetone; 10. glutamate; 11. succinate; 12. carnitine; 13. trimethylamine; 14. creatine; 15. ethanolamine; 16. *o*-acetylcarnitine; 17. choline; 18. carnitine/*sn*-glycero-3-phosphocholine; 19. taurine; 20. glycine; 21. glycerol; 22. threonine; 23. myo-inositol; 24. *sn*-glycero-3-phosphocholine; 25. hydroxyacetone; 26. adenosine/inosine; 27. glucose; 28. uracil; 29. uridine; 30. tyrosine; 31. phenylalanine; 32. niacinamide; 33. hypoxanthine; 34. inosine; 35. adenosine; and 36. formate. Water (4.74–4.88 ppm) and residual methanol (3.34–3.37 ppm) regions were excluded. (c,d) Lipophilic extracts from (c) SC diet and (d) HF chow diet. Vertical intensity is matched between spectra. Main assignments are as follows: 1. cholesterol; 2. fatty acids (FA) (non-ω-3 CH_3_); 3. FA (ω-3 CH_3_); 4. FA ((CH_2_)_*n*_); 5. FA (β-carbon); 6. unassigned (U_1.67_ and U_1.73_); 7. monounsaturated FA; 8. polyunsaturated FA (allylic); 9. unassigned (U_2.25_); 10. FA (α-carbon); 11. unassigned (U_2.38_); 12. linoleic acid; 13. polyunsaturated FA (bis-allylic); 14. phosphatidylethanolamine (tentative assignment); 15. phosphatidylcholine and lysophosphatidylcholine (PC and LPC); 16. unassigned (U_3.39_, U_3.54_, and U_3.66_); 17. sphingomyelin and PC; 18. phospholipids (tentative assignment); 19. triacylglycerols (sn1/sn3); 20. total glycerophospholipids (GPL) (tentative assignment); 21. total GPL (except LPC); 22. triacylglycerols (sn2); and 23. unsaturated FA.

### Effect of High-Fat Diet versus Standard Chow on the Kidney Metabolome

PCA of the spectra obtained from kidney aqueous extracts (Figure 3a) did not show significant separation between SC and HF, whereas the corresponding PLS-DA (Figure 3b) suggested a potential group separation (*Q* ^2^ = 0.49). This was confirmed by a permutation *p*-value of 0.014, a *Q* ^2^_median_ of 0.33, and a k-fold AUROC of 0.85 (Figure 3g,h). Despite the absence of PLS-DA classification power, LV1 loadings analysis (Figure 3e) revealed a high-scoring VIP for the TMA peak, suggesting lower TMA levels in the HF group compared to SC. Other metabolites that presented high VIP scores included glycine, alanine, malonate, and *o*-acetylcarnitine (suggested decreases in HF vs. SC) and *sn*-glycerol-3-phosphocholine (GPC) (suggested increase in HF vs. SC). As described in Table 1, analysis of the ^1^H NMR signal integrals from kidney aqueous extracts showed significant differences in concentrations of 11 identified metabolites and 1 unassigned compound (δ 6.8 ppm, U_6.80_) between SC and HF diets. Thus, for HF relative to SC, there were increased levels of pyruvate, GPC, and uracil, and decreases in alanine, succinate, methionine, DMA, TMA, malonate, *o*-acetylcarnitine, glycine, and the unassigned signal at 6.8 ppm (Table 1 and Figure 4). After FDR correction, there were four signals that maintained significant differences: methionine, DMA, malonate, and U_6.80_.

**Figure 3:**
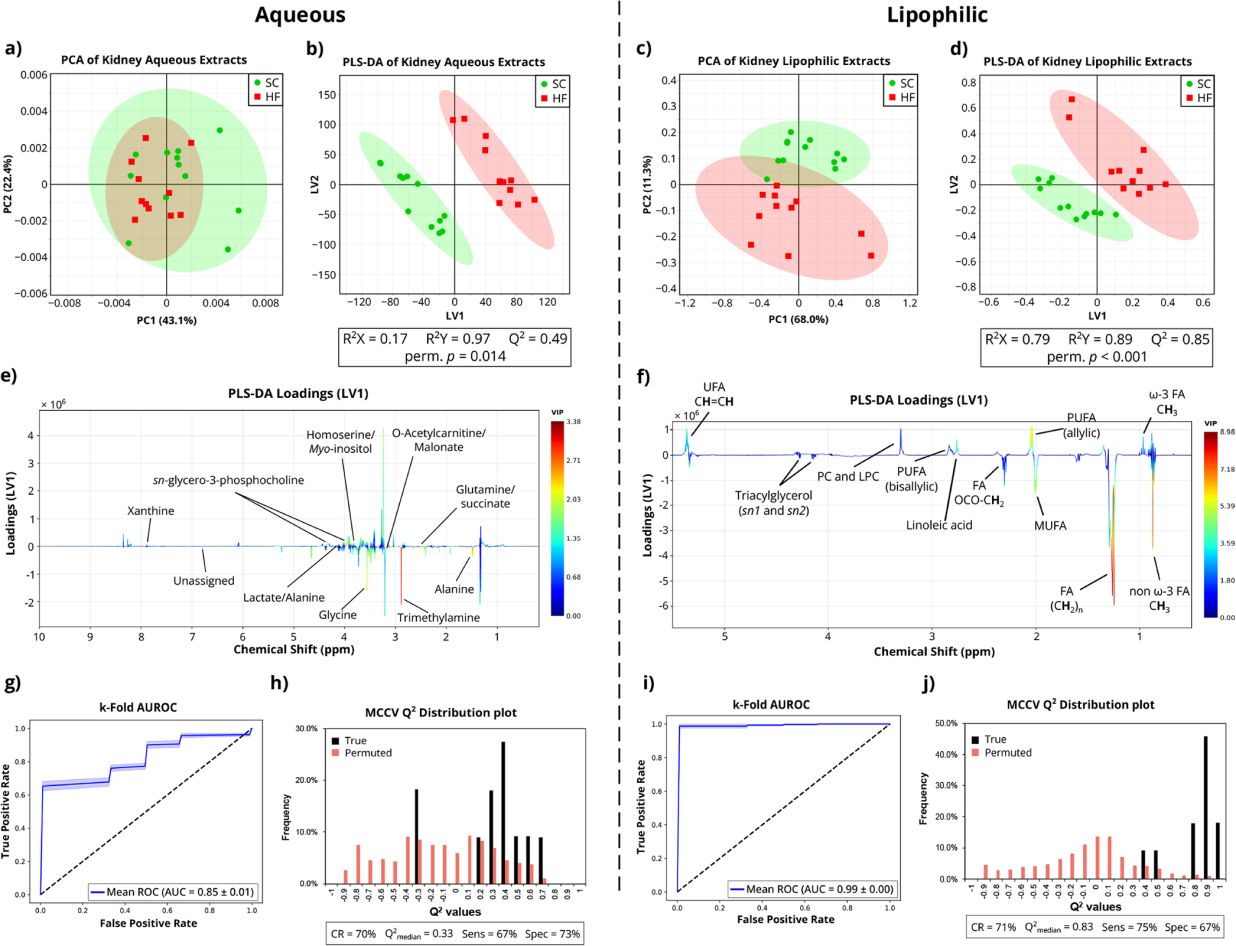
Multivariate statistical analysis and model validation for the ^1^H NMR spectra from aqueous (left) and lipophilic (right) extracts of kidney tissue from mice fed with SC or HF diets. (a-–d) Score scatter plots (95% confidence interval ellipses, standard chow and high-fat diets in green and red, respectively) for (a,c) PCA, with Pareto data scaling, and (b,d) PLS-DA, with UV data scaling used for aqueous model, and Pareto scaling for lipophilic model. Validation parameters (*R* ^2^, *Q* ^2^, and 500 permutation *p*-values) are indicated for each PLS-DA. (e,f) LV1 loadings plots from PLS-DA model in (b) and (d), respectively, with coloring according to VIP. (g,i) AUROC plot, with UV scaling and 5 k-fold (100 permutations each), with 95% confidence interval. (h,j) Plot of true and permutated classes, using MCCV with UV data scaling and 500 permutations.

**Table 1:** Significant Metabolite Variations Measured in Kidney Extracts Between HF and SC Diets, Organized by Their Chemical Shift^a^ ^b^

Metabolite	δ ^1^H in ppm (multiplicity)	Effect Size (ES)(ES Error)	*p* **-**value	FDR adjusted *p*-value
**Aqueous extracts**				
Alanine	1.48 (d)	–1.40 (0.91)	0.006	ns
Pyruvate	2.37 (s)	1.02 (0.87)	0.023 ^n^	ns
Succinate	2.40 (s)	–1.01 (0.87)	0.032	ns
Methionine	2.13 (s)	–1.45 (0.92)	0.002	0.033
Dimethylamine	2.72 (s)	–1.27 (0.90)	0.007	ns
Trimethylamine	2.89 (s)	–2.09 (1.02)	<0.001 ^n^	0.002
Malonate	3.11 (s)	–1.36 (0.91)	<0.001 ^n^	0.019
*O*-Acetylcarnitine	3.19 (s)	–0.99 (0.87)	0.026	ns
Glycine	3.56 (s)	–1.32 (0.90)	0.005	ns
*sn*-Glycerol-3-phosphocholine	4.33 (m)	1.05 (0.87)	0.019	ns
Uracil	5.80 (d)	0.87 (0.86)	0.049	ns
U_6.80_	6.80	–1.50 (0.93)	<0.001 ^n^	0.014
**Lipophilic extracts**				
MUFA	2.00 (m)	–0.98 (0.87)	0.013 ^n^	ns
Linoleic acid	2.76 (t)	2.37 (1.07)	<0.001 ^n^	0.001
PUFA (allylic)	2.04 (m)	2.64 (1.12)	<0.001 ^n^	0.001
UFA	3.35 (m)	1.27 (0.90)	0.008	ns

a All variations were confirmed by student *t*-test with Welch correction (or Mann–Whitney test for those with non-parametric distribution) and adjusted according to Benjamini and Hochberg FDR correction for multiple comparisons.^35^

b ^n^non-parametric test was performed. Abbreviations: ES, effect size (calculated as described in reference;^31^ FA, fatty acid; FDR, false discovery rate; MUFA, monounsaturated FA; ns, non-significant variation; PUFA, polyunsaturated fatty acid; UFA, unsaturated FA; Uδ, unassigned signal at chemical shift δ.

**Figure 4:**
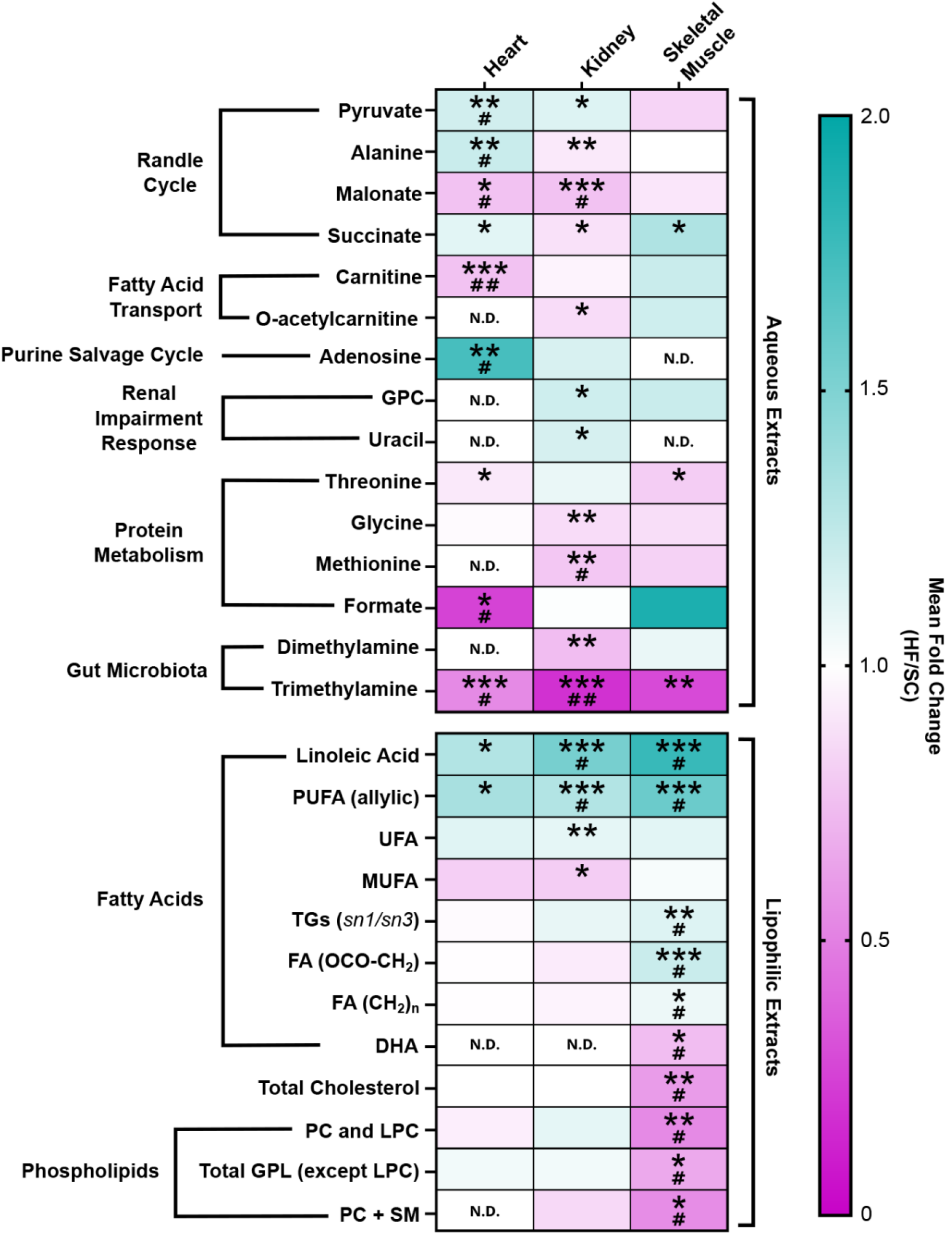
Heatmap showing statistically significant alterations observed between SC and HF diets in aqueous extracts and lipophilic extracts of heart, kidney, and skeletal muscle. Metabolites and lipidic assignments are colored according to their mean fold change (HF levels relative to SC), from minimum (dark pink) to maximum (dark teal) values; N.D. indicates not detectable levels of the metabolite. Statistical significance represented as shown in Table 1 (and Tables S2 and S3), representing Student’s unpaired *t* test with Welch’s correction or Mann–Whitney test for nonparametric (* *p*-value < 0.05; ** *p*-value < 0.01; *** *p*-value < 0.001) and Benjamini and Hochberg FDR correction for multiple comparisons (# *p*-value < 0.05; ## *p*-value < 0.01; ### *p*-value < 0.001). Abbreviations: DHA, docosahexaenoic acid; FA, fatty acid; GPL, glycerophospholipid; GPC, *sn*-glycero-3-phosphocholine; LPC, lysophosphatidylcholine; MUFA, monounsaturated fatty acid; PC, phosphatidylcholine; PUFA, polyunsaturated fatty acid; SM, sphingomyelin; TG, triacylglycerols; and UFA, unsaturated fatty acid.

^1^H NMR signals of lipophilic extracts yielded robust PCA and PLS-DA models, with clear group separation in PCA (∼80% of separation explained) and in PLS-DA (*Q* ^2^ = 0.85) (Figure 3c,d). The strength of the PLS-DA model was confirmed by a permutation *p*-value < 0.001, *Q* ^2^_median_ of 0.83, and a k-fold AUROC of 0.99 (Figure 3i,j). PLS-DA LV1 loadings identified many peaks with relevant VIP values in the separation of HF vs SC, suggesting increases in total unsaturated fatty acids (UFA) driven by contributions from PUFA-allylic and linoleic acid signals, partially offset by decreases in MUFA and non-ω-3 FA signals (Figure 3d). The lipophilic extract integrations mostly confirmed the observations made in the respective loadings, as 4 identified lipid signals were found to be significantly changed in HF relative to SC, namely increased levels of linoleic acid, PUFA (allylic) and UFA, with decreases in MUFA (Table 1 and Figure 4). After FDR correction, differences in linoleic acid and PUFA between HF and SC remained significant.

### Effect of High-Fat Diet versus Standard Chow on the Heart Metabolome

PCA of the ^1^H NMR spectra of heart aqueous extracts did not reveal separation between HF and SC groups (Figure S4a) while PLS-DA suggested a mild group separation (*Q* ^2^ = 0.41) (Figure S4b). Assessment of the PLS-DA model validity confirmed the absence of significant metabolite differences between diet groups; in spite of an AUROC of 0.94, the permutation test failed with a *p* = 0.108 while MCCV yielded a *Q* ^2^_median_ of 0.21 (Figure S4g,h). Despite the absence of group separation in the MVA, differences in water-soluble metabolites were found, namely increased levels of alanine, pyruvate, succinate, adenosine, and unassigned signals at δ 3.86 and 3.90, as well as decreases in TMA, malonate, carnitine, threonine, formate, and unassigned signals at δ 2.27 and 3.61 in HF compared to SC (Table S2 and Figure 4). After FDR correction, seven metabolites in the aqueous extracts maintained significant differences: alanine, pyruvate, TMA, carnitine, adenosine, U_3.86_ and U_3.90_. ^1^H NMR MVA of lipophilic extracts revealed a similar result to that of the aqueous extracts (Figure S4c,d,f,i,j), indicating that, in comparison to the kidney, the effects of HF on the heart metabolome were less pronounced. For the lipophilic extracts, there were significantly higher concentrations of linoleic acid and PUFA (allylic) signals in HF relative to SC (Table S2 and Figure 4), although none retained significance after FDR adjustment.

### Effect of High-Fat Diet versus Standard Chow on the Skeletal Muscle Metabolome

Skeletal muscle aqueous extracts revealed a similar PCA and PLS-DA performance to that of heart aqueous samples, with no resolution by PCA but an apparent distinction between SC and HF diets found by PLS-DA (*Q* ^2^ = 0.45) (Figure S5a,b). As for the heart aqueous samples, the validation presented a reasonable k-fold AUROC of 0.89 but failed the permutation test with *p* = 0.284 and MCCV *Q* ^2^_median_ of 0.2, once again indicating possible model overfitting (Figure S5g,h). The LV1 loading analysis suggested changes in only a few signals: increased concentrations of succinate and decreased levels of TMA in HF relative to SC (Figure S5e). The aqueous fractions presented 5 significant alterations between diets, with increased levels of succinate and an unassigned signal at δ 3.01, and decreased levels of TMA, threonine, and an unassigned signal at δ 5.46 (HF vs. SC) (Table S3). After the FDR *p*-value adjustment for multiple comparisons, all components in the aqueous fraction lost significance.

In contrast, signals from the lipophilic extract of this tissue generated MVA models. PCA presented some separation between groups (∼67% of variance explained in PC1 and PC2), while PLS-DA yielded good separation between groups (1 sample misclassification) and a satisfactory *Q* ^2^ score of 0.49 (Figure S5c,d). Moreover, the PLS-DA model achieved a valid permutation score (*p* = 0.002), as well as high k-fold AUROC (0.87) and MMCV *Q* ^2^_median_ values (0.74) (Figure S5i,j). LV1 loadings analysis revealed a much more diverse set of peaks highlighted with higher VIP scores than what was observed in either the heart or kidney, hinting at a more diverse range of features with possible significant differences. From this loading analysis, we observed a positive separation related to increased levels of linoleic acid, α and β carbons from FA, PUFA (allylic), FA (CH_2_)_*n*_, and FA non-ω-3 CH_3_ in HF relative to SC, and a decrease in the joint signal of phosphatidylcholine (PC) and lysophosphatidylcholine (LPC) (Figure S5f). The loadings analysis of skeletal muscle aqueous and lipophilic extracts recapitulated to a large extent the results of the UVA. In the lipophilic fractions, 18 significant changes between HF and SC were found, with elevated concentrations of backbone chain (CH_2_)_*n*_, α and β carbon signals from FA, linoleic acid, PUFA, and TG (both *sn*1/*sn*3 and *sn*2 signals), and decreases in total cholesterol, docosahexaenoic acid (DHA), PC (together with either LPC or sphingomyelin (SM)), total glycerophospholipid (GPL) (except LPC), an unknown signal attributed to phospholipids, and 4 unassigned signals (U_2.38_, U_2.34_, U_2.38_, and U_2.38_), with all retaining their significance after FDR correction (Table S3 and Figure 4).

### TMA Levels in the Aqueous Fractions

MVA and UVA analyses of the tissue aqueous fractions revealed that TMA was a principal metabolite explaining the separation between HF and SC groups. On this basis, we attempted to estimate TMA levels (μmol per g of tissue) as well as its related metabolites, dimethylamine (DMA) and trimethylamine-*N*-oxide (TMAO), from their ^1^H NMR signals. At the magnetic field strength of the spectrometer (11.7 T), the signals assigned to TMAO showed extensive overlap with those of taurine and glucose, possibly disrupting their importance in the MVA models and preventing integration for UVA. Consequently, TMAO levels were estimated by subtracting the contributions of the overlapping metabolites.

As shown in Figure 5, our estimates of TMA concentrations were concordant with the UVA outputs, indicating a consistent decrease in TMA across the three analyzed tissues of HF versus SC. More importantly, it also showed that while TMA (and DMA in the kidney) were decreased, no significant alterations were detected for TMAO in any of the three tissues.

**Figure 5:**
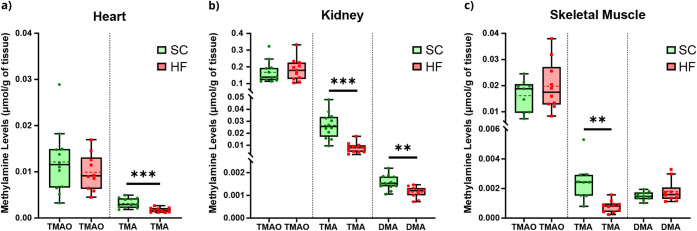
Estimated methylamine levels, normalized by tissue weight, from the effect of SC and HF diets in the lipophilic extracts of the (a) heart, (b) kidney, and (c) skeletal muscle. All graphs represent individual Student’s unpaired *t*-test with Welch’s correction (or Mann–Whitney test for cases where groups failed normality) within each tissue for diet effect, with α=0.05 (* *p*-value < 0.05, ** *p*-value < 0.01, and *** *p*-value < 0.001). Abbreviations: DMA dimethylamine; TMAO trimethylamine *N*-oxide; and TMA trimethylamine.

### Organ Fatty Acid Content and Saturation Profiles

Both MVA and UVA indicated increased lipid signals in HF versus SC, particularly PUFA and linoleic acid. To complement these data, the overall proportions of saturated, monounsaturated, and polyunsaturated fatty acid species (SFA, MUFA, and PUFA, respectively) were assessed, with total tissue FA content estimated from the sum of all FA terminal CH_3_ signals.^38^ The results of this analysis on the kidney, heart, and skeletal muscle lipophilic extracts are shown in Figure 6. Both the kidney and skeletal muscle presented significantly increased percentages of PUFA in the HF group compared to SC (∼39% versus ∼27%, *p*-value < 0.001, and ∼24% versus ∼19%, *p*-value = 0.005, respectively), while in the heart, there were no significant differences (Figure 6b). On the other hand, the MUFA fraction was significantly lower in all three tissues of HF compared to SC mice (∼45% versus ∼54%, ∼48% versus ∼55%, and ∼42% versus ∼49%, for heart, kidney, and muscle, respectively, all with *p*-value < 0.001) (Figure 6c). Meanwhile, the fractions of SFA and ω-3 in all three tissues were not significantly different between HF and SC (Figure 6a,d). While total FA levels in skeletal muscle were doubled in HF relative to SC (∼198 versus ∼93 μmol/g of tissue, *p* = 0.003), they were not significantly different in either heart or kidney (Figure 6e).

**Figure 6:**
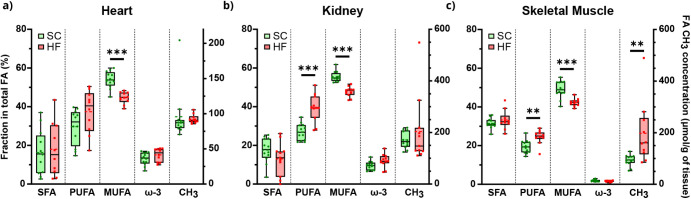
Lipid profiles from the effect of SC and HF diets in the lipophilic extracts of (a) heart, (b) kidney, and (c) skeletal muscle. Fatty acid metrics are in terms of percentage relative to total FA levels (left *y*-axis) as fraction of saturated FA (SFA), polyunsaturated FA (PUFA), monounsaturated FA (MUFA), and ω-3 CH_3_ in each tissue between each diet. Additionally, measure of total FA level variation (right *y*-axis) as concentration of FA CH_3_, normalized for sample weight. All graphs represent individual Student’s unpaired with Welch’s correction (or Mann–Whitney test for cases where groups failed normality), within each tissue for diet effect, with α=0.05 (* *p*-value < 0.05, ** *p*-value < 0.01, and *** *p*-value < 0.001).

## Discussion

In mouse models, MASLD is often induced by placing the animals on a high-fat diet for several weeks. As for the human condition, the development of MASLD is accompanied by systemic changes in adiposity and weight gain. Therefore, it is not surprising that, in addition to the liver, the metabolic states of other tissues are also altered, including some of the major players in lipid oxidation and glucose uptake, such as the skeletal muscle, kidney, and heart. The altered metabolite profiles likely represent changes in available oxidizable substrates resulting from the different diet, such as the increased availability of fatty acids compared to other macronutrients in the high-fat diet, as well as alterations in tissue substrate metabolism resulting from the development of insulin resistance, such as impaired insulin-mediated glucose uptake. To the extent that metabolic perturbations in these extrahepatic tissues influence MASLD progression, their metabolome profiles may be important descriptors of MASLD pathogenesis. Since high-fat feeding per se does not induce the more severe forms of liver injury that are characteristic of MASH, we do not know from these studies whether the observed alterations in extrahepatic tissue metabolite profiles associated with simple MASLD would be the same or different for MASH. We also believe that, while metabolic alterations in extrahepatic tissues may have limited power in predicting MASLD onset and progression in comparison to more liver-specific markers, they could be valuable in anticipating important extrahepatic comorbidities secondary to MASLD, including cardiovascular complications and renal dysfunction.

### NMR Metabolomics for Identifying Alterations in Extrahepatic Tissues in a MASLD Setting

Of the three extrahepatic organs studied, the kidney lipophilic and aqueous metabolomes showed the highest sensitivity toward high-fat feeding and the establishment of simple MASLD—despite there being no significant difference in total kidney FA levels between HF and SC mice. In the PLS-DA of aqueous metabolites, the significant differences in pyruvate, alanine, succinate, malonate, and *o*-acetylcarnitine observed between HF and SC are consistent with alterations in fuel substrate metabolism, possibly related to shifts in carbohydrate and lipid oxidation secondary to insulin resistance. High-fat feeding was also associated with significant increases in kidney GPC and uracil. The accumulation of renal GPC has been linked to organ stress secondary to osmolytic imbalance,^49,50^ while increases in kidney uracil levels have also been shown to associate with streptozotocin-induced diabetes onset in a rodent model as well as with CKD.^51,52^ In the lipophilic fractions, the clear shift in FA distributions toward MUFA in HF versus SC mice—with the linoleic acid component resolved by ^1^H NMR—reflects the high proportion of linoleic acid in the high-fat diet formulation. This was also observed in the lipophilic fractions of the heart and skeletal muscle.

In diet-induced obesity and MASLD, the buildup of ectopic lipid in major extrahepatic sites such as skeletal muscle, heart, and kidney is associated with significant metabolic perturbations in these organs. This, in turn, is anticipated to generate metabolome profiles that are distinct from those of healthy tissues. These profiles can include alterations in FA species resulting from changes in dietary FA composition or modifications in FA profiles of very-low-density lipoprotein (VLDL) exported from the liver. Furthermore, to the extent that dyslipidemia perturbs intermediary metabolism and nutrient fluxes in specific organs, alterations in their endogenous water-soluble metabolites are also anticipated. Finally, intestinal microbiome activity generates distinctive metabolites, such as short-chain fatty acids and products of choline degradation, which contribute to the metabolomes of many tissues. Since alterations in diet can induce significant changes in both the intestinal microbiome^53^ and metabolite^54–56^ profiles, these changes can, in turn, influence tissue metabolomes. Given the diversity of possible factors that can influence the ^1^H NMR tissue metabolome signals, MVA and PLS-DA represent the most efficient and unbiased approaches for classification and identification of the metabolome components that best explain the differences between classes.

Aside from significantly higher total FA—principally in the form of PUFA—the effects of high-fat feeding on the skeletal muscle metabolome were more diminished compared to that of the kidney, suggesting that the impact of high-fat feeding on skeletal muscle intermediary metabolism was more limited compared to the kidney. While the metabolome of heart tissue was overall little affected by high-fat feeding, the higher levels of adenosine are consistent with increased endogenous adenosine generation—a response to cardiomyocellular stressors such as ischemia and inflammation.^57,58^

### Relationship Between High-Fat Feeding, Intestinal Choline Metabolism, and Systemic Metabolic Dysregulation

Both the availability and metabolism of dietary choline play critical roles in the pathophysiology of MASLD and in metabolic perturbations of extrahepatic tissues. The active synthesis of phosphatidylcholine from its dietary precursor is required for hepatic VLDL secretion;^59^ hence, a deficiency in hepatic choline can promote the hepatic retention of TG, thereby potentiating MASLD. Phosphatidylcholine is also an essential component of chylomicrons^60^ and *de novo* synthesis from choline in the epithelium of the small intestine is required for dietary lipid absorption.^61^ High-fat feeding increases the rate of chylomicron synthesis and secretion^62^ thereby placing an increased demand on dietary choline. At the same time, high-fat feeding induces physiological changes in the colonic epithelium that promote microbial choline catabolism to TMA in the lumen.^63^ Thus, in this setting, any dietary choline that escapes chylomicron synthesis has a higher prospect of microbial degradation to TMA and, to a lesser extent, to DMA.

Following absorption, a large proportion of TMA is converted by the liver to TMAO, which is not only a cardiovascular risk biomarker^64^ but also aggravates both MASLD^65^ and diabetic kidney disease.^66^ Our studies showed that while tissue TMAO levels were not different between HF and SC mice, there were consistently lower levels of both TMA and DMA in all the tissues studied. Moreover, MVA identified these metabolites as among the most important features that explained the differences between the tissue metabolomes of HF and SC mice. Given that dietary choline levels were the same for both high-fat and standard chow diets, we hypothesize that the reduced tissue TMA and DMA levels seen in HF compared to those in SC mice may, in part, be explained by the increased demand for choline to sustain higher rates of chylomicron synthesis. Therefore, even if the intestinal microbiota had been conditioned by high-fat feeding to convert choline more efficiently to TMA, there would have been less choline available for this step.

### Increased Ectopic Fat Deposition Levels

During diet-induced MASLD, peripheral tissues face fat overload from dietary lipid sources as well as from the accelerated export of VLDL from the liver. These factors can influence both the total amount and composition of fatty acid moieties within a particular tissue. The pathogenesis of MASLD in the setting of high-fat intake is accompanied by metabolic perturbations in a number of other tissues, some of which may, in turn, influence the progression of MASLD. To date, one of the best-characterized examples is the interaction between the liver and skeletal muscle. Skeletal muscle is among the principal sinks for insulin-mediated whole-body glucose disposal, but this action becomes impaired under hyperlipidemic conditions due to the development of insulin resistance—a condition that is tightly associated with the accumulation of intramyocellular lipid.^67,68^ The resulting chronic hyperglycemia, in turn, promotes hepatic *de novo* lipogenesis.^69^ With a doubling of total FA levels in the skeletal muscle tissue samples, our HF mouse model recapitulated this important aspect of metabolic syndrome. Moreover, this was identified by MVA as the principal feature of the skeletal muscle metabolome that explained the difference between the HF and SC mice. However, an important limitation of our analysis is that it did not resolve intra- and extramyocellular lipid pools, the former being more highly associated with the development of whole-body insulin resistance.^67,68^

In contrast to skeletal muscle, neither heart nor kidney analyses presented evidence of increased lipid burdens in our mouse model, as assessed by total tissue FA levels. Although increased levels of myocardial lipid have been described in both animal models^70^ and humans^71^ and found to be associated with both structural and functional cardiac alterations^71,72^ the relationship with whole-body insulin resistance is less clear compared to that of skeletal muscle lipid. While one study found an association between elevated myocardial lipid and impaired glucose tolerance^73^ other studies found that it was dissociated from both hepatic and peripheral insulin sensitivity.^74,75^

In obese humans, ectopic lipid accumulation in the kidney is a recognized feature and is associated with the development of glomerulopathy and other kidney diseases.^76^ While our model of simple MASLD did not show differences in total kidney FA levels compared with normal chow-fed mice, there were nevertheless significant perturbations in FA and other metabolite profiles, as previously discussed. In mice that were fed a combination of high fat, cholesterol, and fructose for 16 weeks, whereby they developed MASH, kidney steatosis was observed, and this was associated with glomerular hypertrophy, endoplasmic reticulum stress, and fibrosis.^77^ In humans, it is not known whether overt kidney steatosis develops during simple MASLD or appears only once the condition has progressed to MASH.

## Conclusions

The development of simple MASLD in a high-fat feeding mouse model is accompanied by alterations in the metabolomes of the heart, skeletal muscle, and kidney, as analyzed by ^1^H NMR spectroscopy of aqueous and lipophilic tissue extracts. The shifts in tissue metabolome profiles attributable to high-fat feeding recapitulated many, but not all, of the features of metabolic syndrome and MASLD in humans. While the model recreated ectopic lipid accumulation in skeletal muscle, kidney steatosis was not established. However, the kidney metabolome presented the most diverse and significant alterations, suggesting that metabolic activity in this organ was more disrupted by high-fat feeding and MASLD compared to that of the heart and skeletal muscle. In all three tissues, metabolites of microbial choline metabolism contributed strongly to metabolome variations between high-fat- and normal-chow-fed mice, highlighting the strong interaction between dietary choline metabolism and the classification of obesity-related conditions such as MASLD.

While this study provides valuable insights into MASLD implications for the remaining organs and tissues, it is crucial to recognize some important limitations. The observed changes are single-point alterations, and their relationships to metabolic pathways are suggested based on known interactions and literature. NMR-based metabolomics is generally limited to the coverage of relatively abundant tissue metabolites but, on the other hand, offers a holistic view of profile changes in terms of very distinct compound families. This is particularly useful in untargeted studies, where no *a priori* information is known or considered (such as the current one). However, many of the triggers of aberrant lipid metabolism and insulin signaling, such as ceramides and diacylglycerols, are present at levels that are too low to be detected by ^1^H NMR. Hence, determining the status of these key metabolites would require more sensitive analytical approaches, such as targeted LC-MS/MS. Finally, it is important to note that this study only featured male C57BL/6J mice; hence, we do not know to what extent our observations could have been influenced by sex.

Nonetheless, this work serves as a hypothesis-generation strategy and a first step for future research focused on these pathways, which can provide more evidence on the mechanisms taking place in each of the extrahepatic tissues during MASLD progression.

## Supplementary Material



## Data Availability

This data is available on the NIH Common Fund’s National Metabolomics Data Repository (NMDR) website, the Metabolomics Workbench, https://www.metabolomicsworkbench.org, where it has been assigned Project ID PR002295. The data can be accessed directly via its Project DOI: 10.21228/M8ZZ54. This work is supported by the Metabolomics Workbench/National Metabolomics Data Repository (NMDR) (grant # U2C-DK119886), the Common Fund Data Ecosystem (CFDE) (grant # 3OT2OD030544), and the Metabolomics Consortium Coordinating Center (M3C) (grant # 1U2C-DK119889).

## Notes

Complete list of metabolite assignments in all tissues. Table S2. Significantly altered metabolites in heart extracts. Table S3. Significantly altered metabolites in skeletal muscle extracts. Figure S1. Animal model parameters. Figure S2. Heart representative NMR spectra. Figure S3. Skeletal muscle representative NMR spectra. Figure S4. Heart MVA results. Figure S5. Skeletal muscle MVA results (PDF)
