# Supplementary material for: Impact of High-Fat Diet-induced Metabolic Dysfunction-associated Steatotic Liver Disease on Heart, Kidney, and Skeletal Muscle Metabolomes in Wild-Type Mice
Source: J Proteome Res. 2025 Apr 13;24(5):2491–504. doi: 10.1021/acs.jproteome.5c00040 (PMC12820970; doi:10.1021/acs.jproteome.5c00040)
Supplement: Supplementary file 1 [file pr5c00040_si_001.pdf]

Supporting Information to manuscript:

Impact of High Fat Diet-induced Metabolic Dysfunction-associated Steatotic Liver Disease on Heart, Kidney and Skeletal Muscle Metabolomes in Wild-type Mice

João G. Silva[1,2,3,5,*], Ludgero Tavares[1,3], Getachew D. Belew[1,4], João A. Rodrigues[5], Rita Araújo[5], Ana M. Gil[5,#] and John G. Jones[1,#,*]

[1] University of Coimbra, Institute for Interdisciplinary Research (III-UC), Centre for Innovative Biomedicine and Biotechnology (CIBB), Metabolism, Aging and Disease, Cantanhede, Portugal

[2] University of Coimbra, Institute for Interdisciplinary Research (III-UC), Doctoral Programme in Experimental Biology and Biomedicine (PDBEB), Coimbra, Portugal

[3] Associação Cognitária Vasco da Gama (ACVG), Vasco da Gama Research Centre (CIVG), Coimbra, Portugal

[4] Ohio University, Department of Biomedical Sciences, Heritage College of Osteopathic Medicine, Athens, Ohio, United States

[5] University of Aveiro, Department of Chemistry and CICECO-Aveiro Institute of Materials, Aveiro, Portugal

# these authors contributed equally to this work.

***Address for Correspondence:** John G. Jones, Center for Neurosciences and Cell Biology, UC-Biotech, Biocant Park; Núcleo 8, Lote 4, 3060-197, Cantanhede, Portugal. email: john.griffith.jones@gmail.com

or

João G. Silva, Center for Neurosciences and Cell Biology, UC-Biotech, Biocant Park; Núcleo 8, Lote 4, 3060-197, Cantanhede, Portugal. email: jgsilva@cnc.uc.pt

Table S1. Complete list of metabolite assignments in all tissues.

Table S2. Significantly altered metabolites in heart extracts.

Table S3. Significantly altered metabolites in skeletal muscle extracts.

Figure S1. Animal model parameters.

Figure S2. Heart representative NMR spectra.

Figure S3. Skeletal muscle representative NMR spectra.

Figure S4. Heart MVA results.

Figure S5. Skeletal muscle MVA results.

Table S1

Table S1. List of compounds identified in the ^{1}H NMR spectra of mice kidney, heart and skeletal muscle aqueous and lipophilic extracts, displayed into two columns. [a] signal integrated, within the full compound spin system; [b] Tissue in which metabolite was detected: K kidney; H heart; M skeletal muscle; [c] metabolite ID found in the Human Metabolome Database (HMDB). Abbreviations: DHA docosahexaenoic acid; FA fatty acid; GPL glycerophospholipid; GTP guanosine triphosphate; IMP inosine monophosphate; LPC lysophosphatidylcholine; MUFA monounsaturated fatty acid; PC phosphatidylcholine; PE phosphatidylethanolamine; PUFA polyunsaturated fatty acid; SM sphingomyelin; TMAO trimethylamine-N-oxide. s singlet; d doublet; t triplet; m multiplet; br broad; † Tentative of assignment.

Metabolite	δ ^{1}H in ppm [a] (multiplicity)	Tissue [b]	HMDB ID [c]	Metabolite	δ ^{1}H in ppm [a] (multiplicity)	Tissue [b]	HMDB ID [c]
**Aqueous extracts**							
1,3-Dimethylurate	3.33 (s)	M	HMDB0001857	IMP	K: 8.56 (s)<br>M: 6.15 (d)	K M	HMDB0000175
2-Hydroxyisobutyrate	1.34 (s)	K	HMDB0242161	Inosine	6.10 (d)	K H M	HMDB0000195
2-Phosphoglycerate	4.49 (dt)	M	HMDB0000362	Isobutyrate	1.07 (d)	K M	HMDB0001873
3-Hydroxybutyrate	1.20 (d)	K H M	HMDB0000011	Isoleucine	1.01 (d)	K H M	HMDB0000172
Acetate	1.91 (s)	K H M	HMDB00042	Lactate	1.33 (d)	K H M	HMDB0000190
Acetoacetate	2.27 (s)	M	HMDB0000060	Leucine	0.96 (t)	K H M	HMDB0000687
Acetone	2.21 (s)	K H M	HMDB0001659	Lysine	1.73 (tt)	M	HMDB0000182
Adenosine	6.08 (d)	K H	HMDB0000050	Malonate	3.11 (s)	K H M	HMDB0000691
ADP	8.51 (s)	M	HMDB0001341	Methionine	2.13 (s)	K M	HMDB0000696
Alanine	1.48 (d)	K H M	HMDB0000161	*Myo*-Inositol	3.62 (t)	K	HMDB0000211
AMP	8.60 (s)	K H M	HMDB0000045	NAD^+ †	8.43 (s)	M	HMDB0000902
Aspartate	2.8 (dd)	K	HMDB0000191	Niacinamide	8.94 (dd)	K H M	HMDB0001406
ATP	8.53 (s)	M	HMDB0000538	*O*-Acetylcarnitine	3.19 (s)	K M	HMDB0000201
ATP/ADP	6.15 (d/d)	K H	—	Ornithine	3.06 (t)	M	HMDB0000214
Betaine †	3.90 (s)	M	HMDB0000043	Phenylalanine	7.38 (m)	K M	HMDB0000159
Carnitine	2.42 (dd)<br>H: 3.23 (s)	K H M	HMDB0000062	Phosphocholine	3.21 (s)	H M	HMDB0001565
Choline	3.20 (s)	K H M	HMDB0000097	Phosphocreatine †	3.95 (s)	M	HMDB0001511
Creatine	3.03 (s)	K H M	HMDB0000064	Proline	4.13 (dd)	M	HMDB0000162
Creatinine	3.04 (s)	K M	HMDB0000562	Propylene Glycol	1.14 (d)	K	HMDB0001881
Dimethylamine †	2.72 (s)	K M	HMDB0000087	Pyroglutamate	K: 4.18 (dd)<br>M: 2.40 (m)	K M	HMDB0000267
Ethanol	1.18 (t)	H M	HMDB0000108	Pyruvate	2.37 (s)	K H M	HMDB0000243
Ethanolamine	3.14 (t)	K M	HMDB0000149	Sarcosine †	2.75 (s)	H M	HMDB0000271

Formate	8.45 (s)	K H M	HMDB00142	Serine	3.97 (m)	M	HMDB0000187
Fumarate	6.52 (s)	K M	HMDB0000134	*sn*-Glycero-3-phosphocholine	K: 4.33 (m)<br>M: 3.22 (s)	K M	HMDB0000086
Glucose	5.22 (d)	K H M	HMDB0000122	Succinate	2.40 (s)	K H M	HMDB0000254
Glucose-6-phosphate †	4.03 (m)	M	HMDB0001401	Taurine	3.42 (t)	K H M	HMDB0000251
Glutamate	2.35 (m)	H K	HMDB0003339	Threonine	3.58 (d)	K H M	HMDB0000167
Glutamine	2.44 (m)	H M	HMDB0000641	TMAO †	3.26 (s)	M	HMDB0000925
Glycerol	3.56 (dd)<br>H: 3.65 (dd)	K H M	HMDB0000131	Trimethylamine	2.89 (s)	K H M	HMDB0000906
Glycine	3.56 (s)	K H M	HMDB0000123	Tyrosine	6.90 (m)<br>H: 7.20 (m) †	K H M	HMDB0000158
GTP	5.95 (d)	K	HMDB0001273	UMP	8.10 (d)	K	HMDB0000288
Guanosine	8.00 (s)	K	HMDB0000133	Uracil	5.80 (d)	K	HMDB0000300
Histamine/Histidine	7.08 (s/s)	K	HMDB0000870/<br>HMDB0000177	Uridine	K: 5.91 (dd)<br>H: 7.88 (d) †	K H	HMDB0000296
Hydroxyacetone	4.38 (s)	K	HMDB0006961	Valerate †	2.19 (t)	M	HMDB0000892
Hypoxanthine	8.21 (s)	K H	HMDB0000157	Valine	1.04 (d)	K H M	HMDB0000883
**Lipophilic extracts**							
All GPL	4.37 (br)	K H	—	Plasmalogens †	5.90 (d)	K H M	—
DHA †	2.42 (br)	M	HMDB0002183	PC + SM	3.75 (br)	K M	—
FA $(CH_2)_n$	1.27 (m)	K H M	—	PUFA	2.81 (m)	K H M	—
FA (α carbon)	2.30 (m)	K H M	—	PUFA (allylic)	2.04 (m)	K H M	—
FA (β carbon)	1.60 (m)	K H M	—	Saturated FA	0.87 (t)	K H M	—
Linoleic Acid	2.76 (t)	K H M	HMDB0000673	SM	5.67 (m)	K H	—
MUFA (allylic)	2.00 (m)	K H M	—	Total Cholesterol	0.67 (s)	K H M	—
PC and LPC	3.30 (br)	K H M	—	Triacylglycerols (*sn1*/*sn3*)	4.14/4.28 (d/d)	K H M	—
Unidentified Phospholipids	3.98 (br)	K H M	—	Triacylglycerols (*sn2*)	5.25 (m)	K H M	—
Total GPL (except LPC)	5.20 (br)	K H M	—	Unsaturated FA	3.35 (m)	K H M	—
PE †	3.17 (br)	K	HMDB0060501	ω-3 FA CH_3	0.97 (t)	K H M	—

Table S2

Table S2. Significant metabolite variations detected in heart extracts between HF diet and SC diets, organised by their chemical shift. All variations were confirmed by t-student test with Welch correction (or Mann-Whitney test for those with non-parametric distribution), and adjusted according to Benjamini & Hochberg FDR correction for multiple comparisons [35]. ^{n} non-parametric test was performed. Abbreviations: ES effect size (calculated as described in reference [31]; FDR false discovery rate; ns non-significant variation; PUFA polyunsaturated fatty acid; U_{δ} unassigned signal at chemical shift δ.

Metabolite	δ ^{1}H in ppm (multiplicity)	Effect Size (ES) (ES Error)	*p*-value	FDR-adjusted *p*-value
**Aqueous extracts**				
Alanine	1.48 (d)	1.34 (0.93)	0.006	0.034
Pyruvate	2.37 (s)	1.48 (0.95)	0.005	0.034
Succinate	2.40 (s)	0.98 (0.89)	0.041	ns
Trimethylamine	2.89 (s)	-1.77 (0.99)	< 0.001	0.009
Malonate	3.11 (s)	-1.16 (0.91)	0.011	ns
Carnitine	3.23 (s)	-2.08 (1.04)	< 0.001	0.003
Threonine	3.58 (d)	-0.95 (0.89)	0.035	ns
Adenosine	6.08 (d)	1.61 (0.96)	0.005	0.034
Formate	8.45 (s)	-1.14 (0.90)	0.012	ns
$U_{2.27}$	2.27	-0.91 (0.88)	0.038	ns
$U_{3.61}$	3.61	-1.17 (0.91)	0.014	ns
$U_{3.86}$	3.86	1.40 (0.94)	0.005	0.034
$U_{3.90}$	3.90	1.55 (0.96)	0.006	0.034
**Lipophilic extracts**				
PUFA (allylic)	2.04 (m)	1.04 (0.89)	0.032 ^{b}	> 0.05
Linoleic acid	2.76 (t)	1.20 (0.91)	0.023 ^{b}	> 0.05

Table S3

Table S3. Significant metabolite variations detected in skeletal muscle extracts between HF diet and SC diets, organised by their chemical shift. All variations were confirmed by t-student test with Welch correction (or Mann-Whitney test for those with non-parametric distribution), and adjusted according to Benjamini & Hochberg FDR correction for multiple comparisons [35]. ^{n} non-parametric test was performed. Abbreviations: ES effect size (calculated as described in reference [31]; FDR false discovery rate; ns non-significant variation; DHA docosahexaenoic FA fatty acid; GPL glycerophospholipid; LPC lysophosphatidylcholine; PC phosphatidylcholine; PUFA polyunsaturated FA; SM sphingomyelin; TG Triacylglycerols.

**Metabolite**	**δ ^{1}H in ppm (multiplicity)**	**Effect Size (ES) (ES Error)**	***p*-value**	**FDR-adjusted *p*-value**
**Aqueous**				
Succinate	2.40 (s)	1.14 (0.97)	0.023	ns
Trimethylamine	2.89 (s)	-2.10 (1.12)	0.002	ns
Threonine	3.58 (d)	-1.08 (0.96)	0.031 ^{n}	ns
$U_{3.01}$	3.01	1.11 (0.97)	0.026	ns
$U_{5.46}$	5.46	-1.32 (0.99)	0.015	ns
**Lipophilic**				
Total cholesterol	0.67 (s)	-1.56 (0.96)	0.001	0.006
FA $(CH_2)_n$	1.27 (m)	1.11 (0.90)	0.016	0.030
FA (β CH_2)	1.60 (m)	1.75 (0.99)	< 0.001	0.004
PUFA (allylic)	2.04 (m)	2.39 (1.10)	< 0.001	< 0.001
FA (α CH_2)	2.30 (m)	1.81 (0.99)	< 0.001	0.003
DHA	2.42 (br)	-1.02 (0.89)	0.0236	0.039
Linoleic acid	2.76 (t)	2.61 (1.14)	< 0.001 ^{n}	0.003
PC and LPC	3.30 (br)	-1.17 (0.91)	0.010	0.024
PC + SM	3.75 (br)	-1.13 (0.90)	0.023 ^{n}	0.039
Unidentified Phospholipids	3.98 (br)	-1.42 (0.94)	0.004	0.011
TGs (*sn1*/*sn3*)	4.14/4.28 (d/d)	1.39 (0.93)	0.004	0.010
Total GPL (except LPC)	5.20 (br)	-1.49 (0.95)	0.002	0.006
TG (*sn2*)	5.25 (m)	1.99 (1.02)	< 0.001 ^{n}	0.003
$U_{1.67}$	1.67	-1.23 (0.91)	0.008	0.019
$U_{2.34}$	2.34	-1.76 (0.99)	< 0.001	0.003
$U_{2.38}$	2.38	-1.10 (0.90)	0.0146	0.029
$U_{3.66}$	3.66	-0.97 (0.89)	0.013 ^{n}	0.029

Figure S1

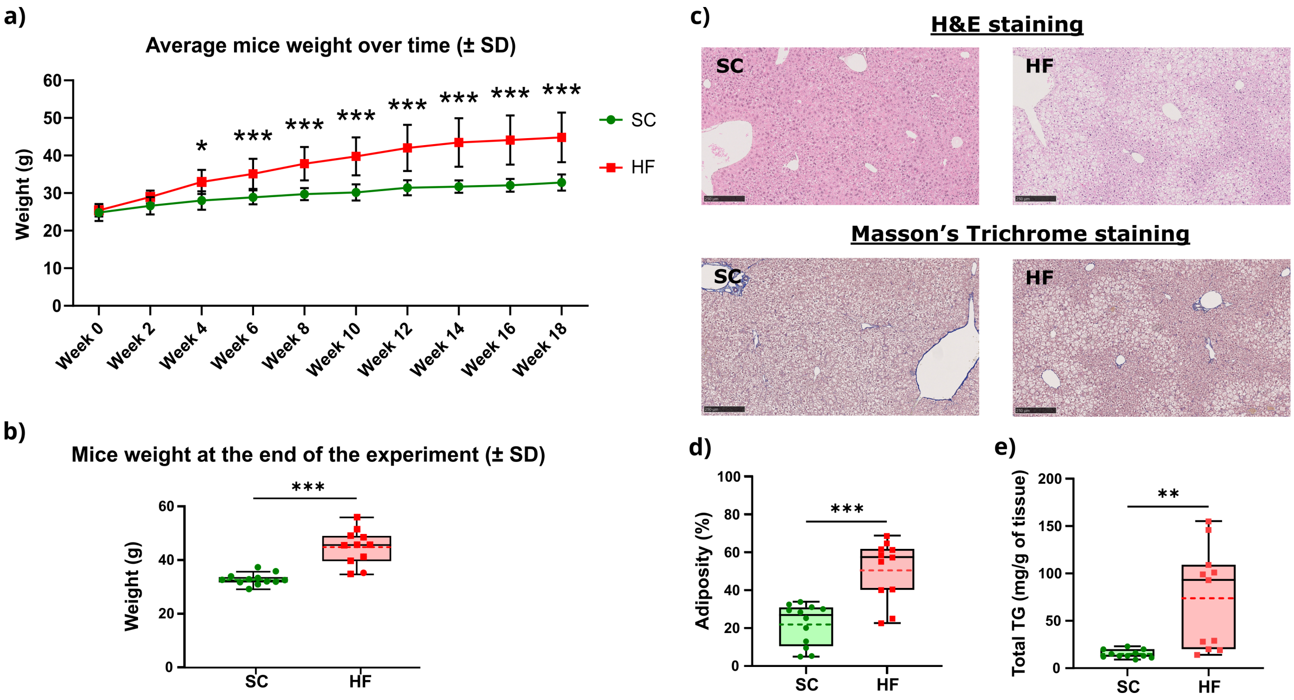

Figure S1. Animal model parameters from the effect of SC and HF diets for 18 weeks. (**a**) Body weight changes over the duration of the experiment. Two-way ANOVA reported significant differences related to diet type and experiment duration (*p*-value < 0.001 for all). * significant differences between SC and HF given by the Šídák's multiple comparisons test, at each given time point, starting at week 4 (* *p*-value = 0.016) and until week 18 (*** *p*-value < 0.001). (**b**) Final mice weight at the end of the experiment. (**c**) Liver histology using hematoxylin and eosin staining and Masson's trichrome stain. (**d**) Body weight fraction (**e**) Liver TG levels. (**b-e**) Student's unpaired t-test with Welch correction (* *p*-value < 0.05, ** *p*-value < 0.01, *** *p*-value < 0.001).

Figure S2

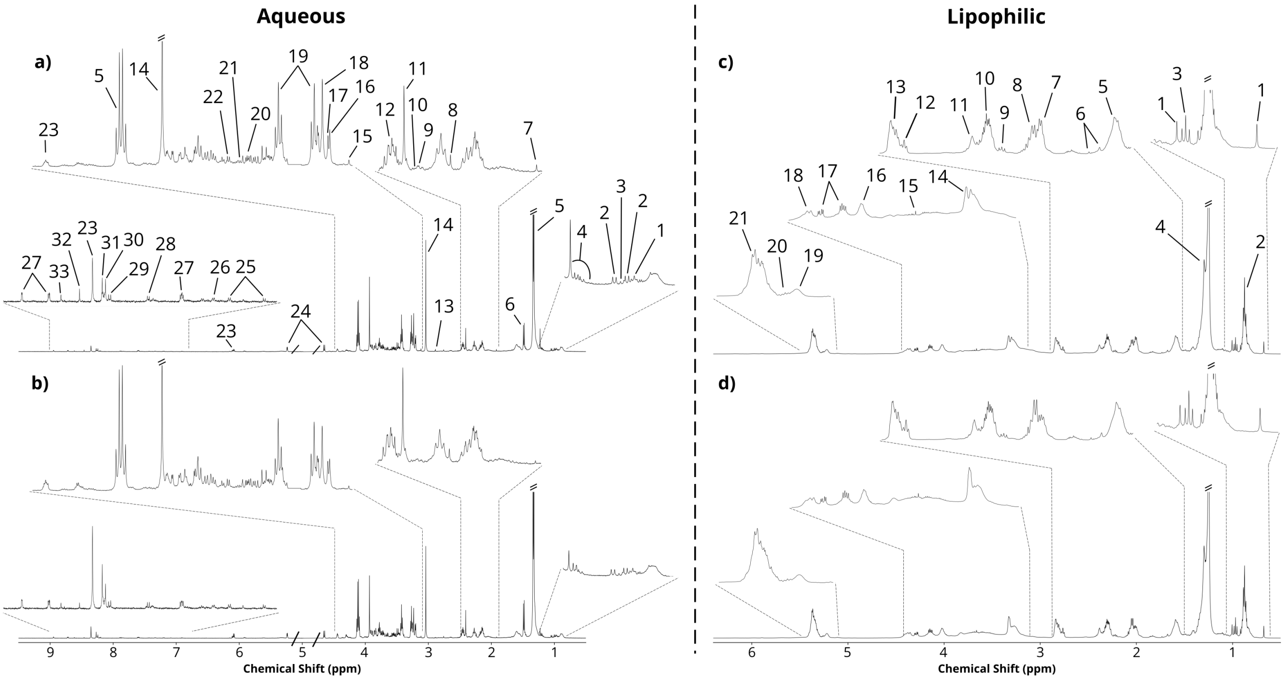

Figure S2. Typical 500 MHz ^{1}H NMR spectra obtained for mice heart extracts. (**a**,**b**) Aqueous extracts from (**a**) SC diet and (**b**) HF diet. Vertical intensity is matched between spectra. Main assignments are as follows: 1. leucine; 2. valine; 3. isoleucine; 4. 3-hydroxybutyrate and ethanol; 5. lactate; 6. alanine; 7. acetate; 8. acetone; 9. glutamate; 10. pyruvate; 11. succinate; 12. glutamine; 13. trimethylamine; 14. creatine; 15. malonate; 16. choline; 17.phosphocholine; 18; carnitine; 19. taurine; 20. glycine; 21. threonine; 22. glycerol; 23. adenosine/inosine; 24. glucose; 25. tyrosine; 26. phenylalanine; 27. niacinamide; 28. uridine; 29. hypoxanthine; 30. inosine; 31. adenosine; 32. formate; 33. AMP. Water (4.70 – 4.98 ppm) and residual methanol (3.32 - 3.38 ppm) regions were excluded. (**c**,**d**) Lipophilic extracts from (**c**) SC diet and (**d**) HF diet. Vertical intensity is matched between spectra. Main assignments are as follows: 1. cholesterol; 2. fatty acids (FA) (non-ω-3 CH_3); 3. FA (ω-3 CH_3); 4. FA ($(CH_2)_n$); 5. 10. FA (β-carbon); 6. unassigned ($U_{1.67}$ and $U_{1.73}$); 7. monounsaturated FA; 8. polyunsaturated FA (allylic); 9. unassigned ($U_{2.25}$); 10. FA (α-carbon); 11. unassigned ($U_{2.38}$); 12. linoleic acid; 13. polyunsaturated FA (*bis*-allylic); 14. phosphatidylcholine and lysophosphatidylcholine (PC and LPC); 15. unassigned ($U_{3.66}$); 16. unassigned ($U_{4.04}$); 17. triacylglycerol (*sn1*/*sn3*); 18. total GPL (tentative assignment); 19. total GPL (except LPC); 20. triacylglycerol (*sn2*); 21. unsaturated FA.

Figure S3

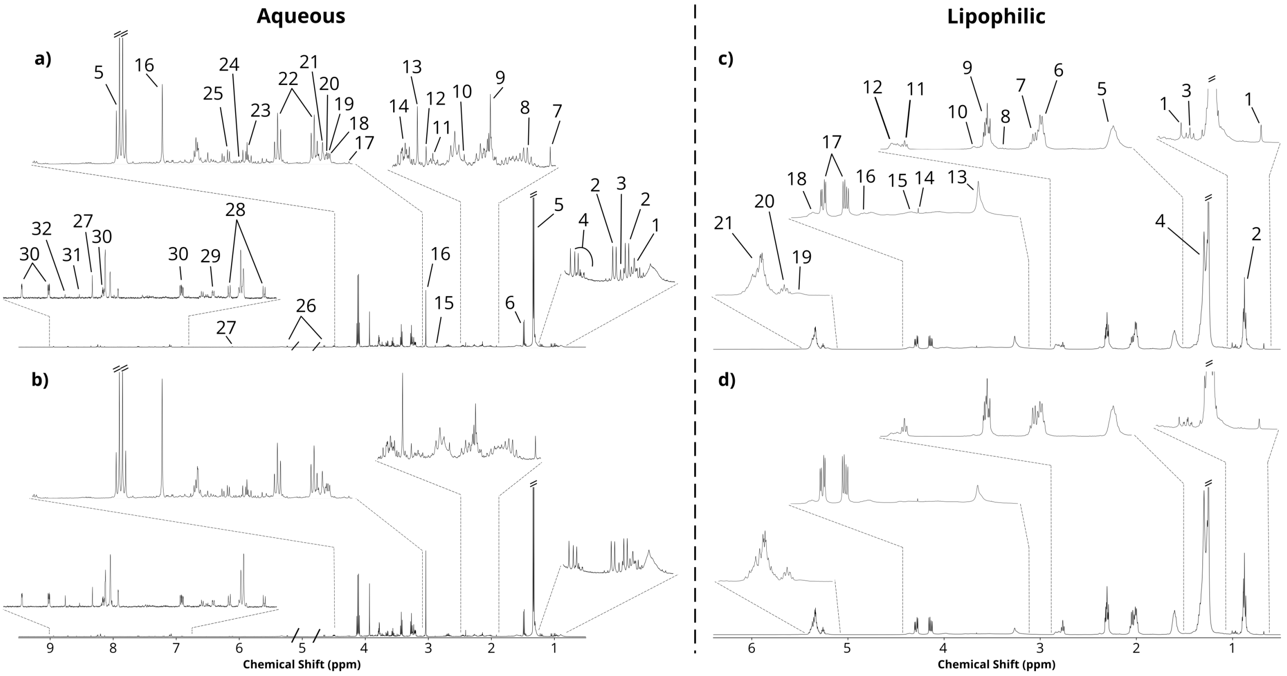

Figure S3. Typical 500 MHz ^{1}H NMR spectra obtained for mice skeletal muscle extracts. (**a**,**b**) Aqueous extracts from (**a**) SC diet and (**b**) HF diet. Vertical intensity is matched between spectra. Main assignments are as follows: 1. leucine; 2. valine; 3. isoleucine; 4. 3-hydroxybtutyrate and ethanol; 5. lactate; 6. alanine; 7. acetate; 8. proline; 9.methionine; 10. acetone; 11. glutamate; 12; pyruvate; 13. succinate; 14. glutamine; 15. trimethylamine; 16. creatine; 17. malonate; 18. *O*-acetylcarnitine; 19. choline; 20. phosphocholine; 21. carnitine; 22. taurine; 23. glycine; 24. threonine; 25. glycerol; 26. glucose; 27. adenosine/inosine; 28. tyrosine; 29. phenylalanine; 30. niacinamide; 31. formate; 32. IMP. Water (4.73 - 4.90 ppm) and residual methanol (3.34 - 3.37 ppm) regions were excluded. (**c**,**d**) Lipophilic extracts from (**c**) SC diet and (**d**) HF diet. Vertical intensity is matched between spectra. Main assignments are as follows: 1. cholesterol; 2. fatty acids (FA) (non-ω-3 CH_3); 3. FA (ω-3 CH_3); 4. FA ($(CH_2)_n$); 5. FA (β-carbon); 6. monounsaturated FA; 7. polyunsaturated FA (allylic); 8. unassigned ($U_{2.25}$); 9. FA (α-carbon); 10. unassigned ($U_{2.38}$); 11. linoleic acid; 12. polyunsaturated FA (*bis*-allylic); 13. phosphatidylcholine and lysophosphatidylcholine (PC and LPC); 14. unassigned ($U_{3.66}$); 15. sphingomyelin and PC; 16. phospholipids (tentative assignment); 17. triacylglycerol (*sn1*/*sn3*); 18. total GPL (tentative assignment); 19. total GPL (except LPC); 20. triacylglycerol (*sn2*); 21. unsaturated FA.

Figure S4

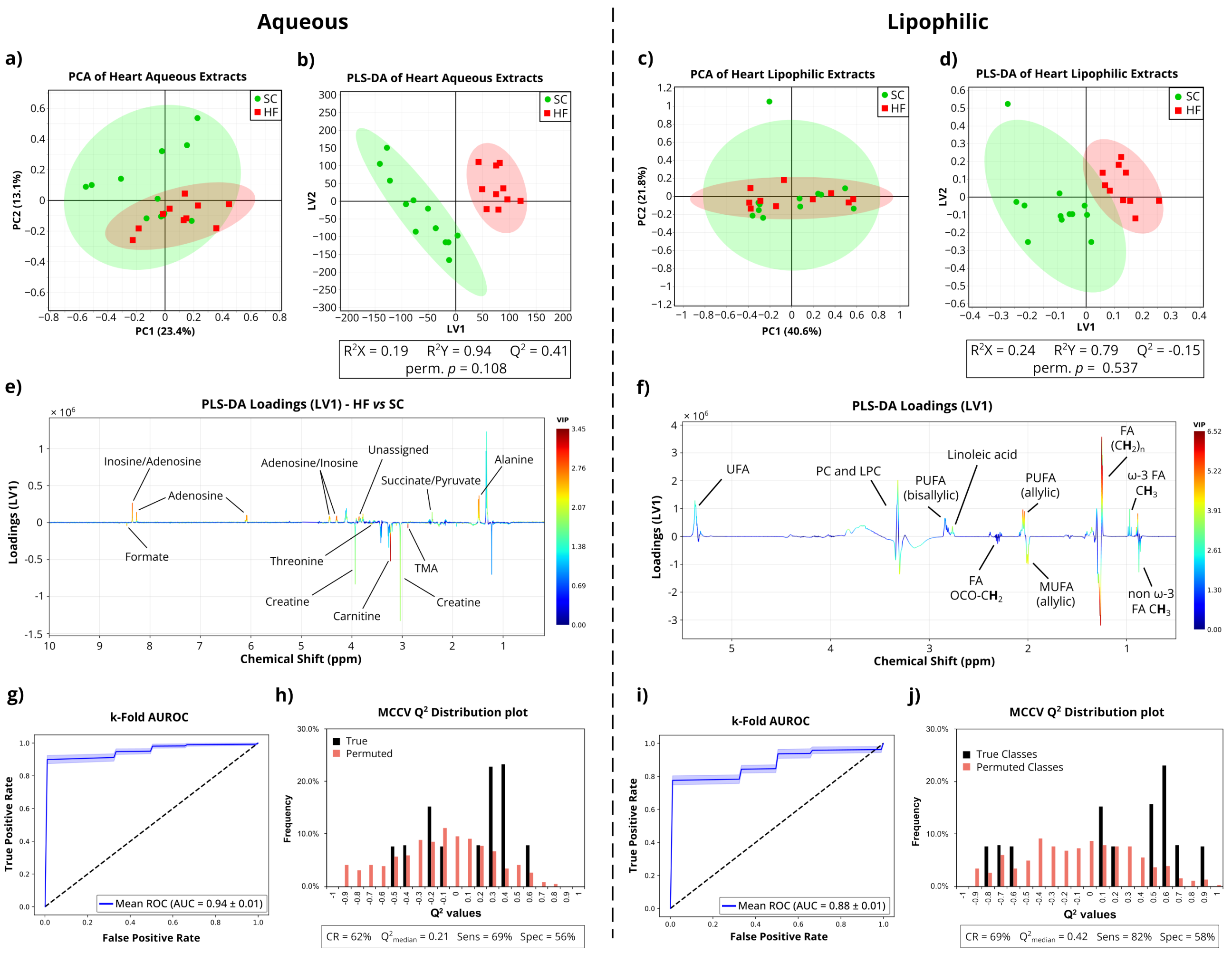

Figure S4. Multivariate statistical analysis and model validation for ^{1}H NMR spectra from aqueous (left) and lipophilic (right) extracts of heart tissue from mice fed with SC and HF diets. (**a-d**) Score scatter plots (95% confidence interval ellipses, SC and HF diets in green and red, respectively) for (**a**,**c**) PCA, with Pareto data scaling, and (**b**,**d**) PLS-DA, with UV data scaling used for aqueous model, and Pareto scaling for lipophilic model. Validation parameters (R^2, Q^2, and 500 permutations *p*-value) are indicated for each PLS-DA. (**e**,**f**) LV1 loadings plots from PLS-DA model in (**b**) and (**d**), respectively, with colouring according to VIP. (**g**,**i**) AUROC plot, with UV scaling and 5 k-fold (100 permutations each), with 95% confidence interval. (**h**,**j**) Plot of true and permutated classes, using MCCV with UV data scaling and 500 permutations.

Figure S5

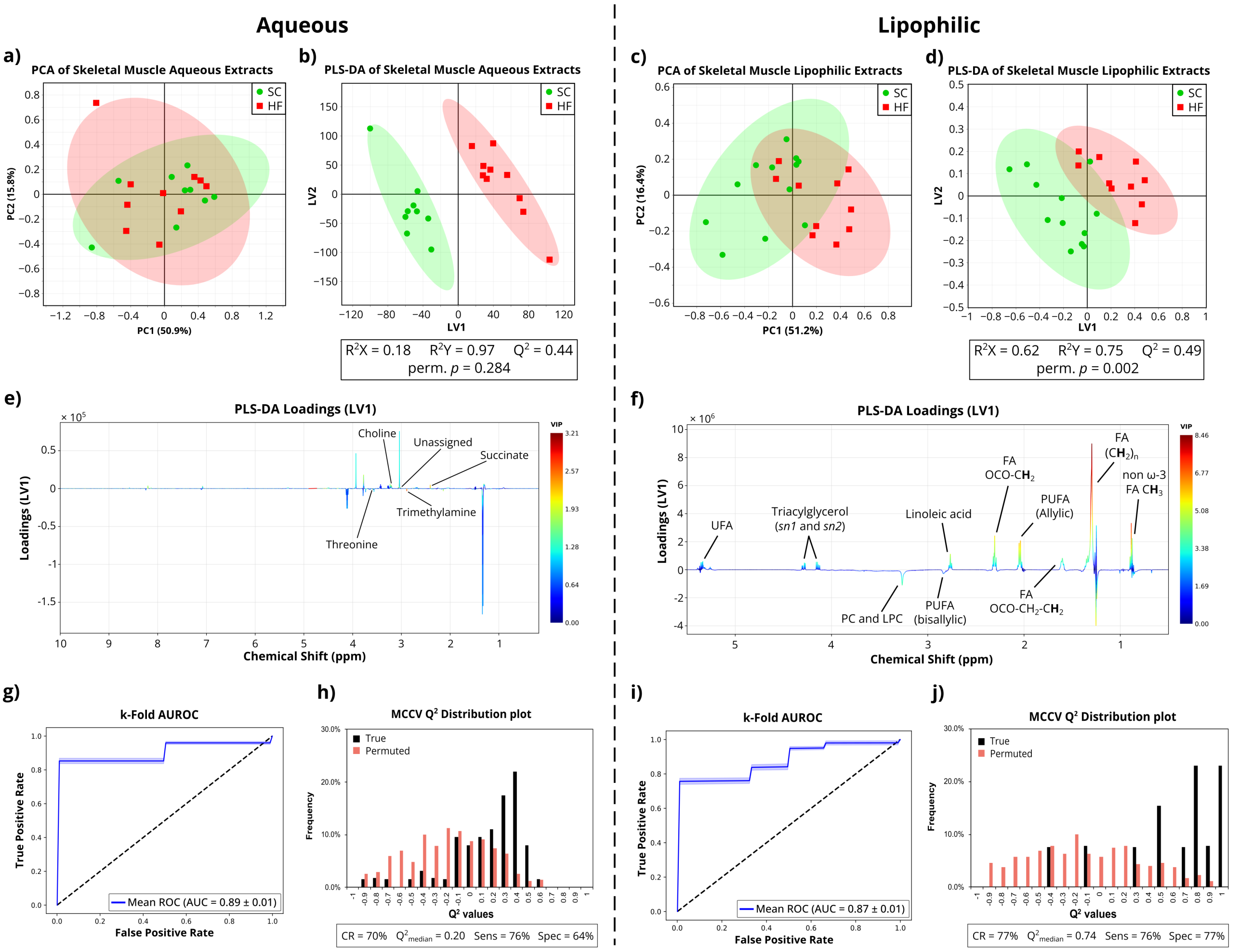

Figure S5. Multivariate statistical analysis and model validation for ^{1}H NMR spectra from aqueous (left) and lipophilic (right) extracts of skeletal muscle tissue from mice fed with SC and HF diets. (**a-d**) Score scatter plots (95% confidence interval ellipses, SC and HF diets in green and red, respectively) for (**a**,**c**) PCA, with Pareto data scaling, (**b**,**d**) PLS-DA, with UV data scaling used for aqueous model, and Pareto scaling for lipophilic model. Validation parameters (R^2, Q^2, and 500 permutations *p*-value) are indicated for each PLS-DA. (**e**,**f**) LV1 loadings plots from PLS-DA model in (**b**) and (**d**), respectively, with colouring according to VIP. (**g**,**i**) AUROC plot, with UV scaling and 5 k-fold (100 permutations each), with 95% confidence interval. (**h**,**j**) Plot of true and permutated classes, using MCCV with UV data scaling and 500 permutations.
